# Considering ivermectin for treatment of schistosomiasis

**DOI:** 10.1007/s00436-024-08178-1

**Published:** 2024-04-09

**Authors:** Jacob Golenser, Ida Birman, Daniel Gold

**Affiliations:** 1 Department of Microbiology and Molecular Genetics, Kuvin Center for the Study of Infectious and Tropical Diseases, The Hebrew University - Hadassah Medical Center, Jerusalem, Israel; 2 Department of Clinical Microbiology and Immunology, Faculty of Medicine, Tel Aviv University, Tel Aviv, Israel

**Keywords:** Ivermectin, Praziquantel, Schistosomiasis, *Schistosoma mansoni*, Parasites, Treatment

## Abstract

Because of recent reports of praziquantel resistance in schistosome infections, there have been suggestions to employ ivermectin as a possible alternative, especially as its chemical composition is different from that of praziquantel, so cross-resistance is not expected. In order to ascertain possible damage and elimination of worms, we used ivermectin by oral gavage in infected mice, at a high dose (30.1 mg/kg, bordering toxicity). We also tested the efficacy of the drug at various times postinfection (PI), to check on possible effect on young and mature stages of the parasites. Thus, we treated mice on days 21 and 22 or on days 41 and 42 and even on days 21, 22, 41, and 42 PI. None of the treatment regimens resulted in cure rates or signs of lessened pathology in the mice. We also compared the effect of ivermectin to that of artemisone, an artemisinin derivative which had served us in the past as an effective anti-schistosome drug, and there was a stark difference in the artemisone’s efficacy compared to that of ivermectin; while ivermectin was not effective, artemisone eliminated most of the worms, prevented egg production and granulomatous inflammatory response. We assume that the reported lack of activity of ivermectin, in comparison with praziquantel and artemisinins, originates from the difference in their mode of action. In wake of our results, we suggest that ivermectin is not a suitable drug for treatment of schistosomiasis.

## Introduction

Schistosomiasis is an acute and chronic parasitic disease caused by blood flukes (*Trematode* worms) of the genus *Schistosoma.* Estimates show that at least 251.4 million people required preventive treatment in 2021. The number of deaths due to schistosomiasis is difficult to estimate because of its hidden pathologies such as liver and kidney failure, bladder cancer, and ectopic pregnancies due to female genital pathology. However, the mortality related to schistosomiasis is estimated at about 12,000 deaths per year. These figures are likely underestimated and need to be reassessed (WHO 2023). Schistosomiasis in humans is caused by five species of schistosomes mainly by *S. mansoni*, *S. japonicum*, and *S. haematobium*. The severe symptoms are caused by immunopathological response to the eggs of the helminths (Coutinho 1961; Mawa et al. 2021).

**Scheme 1:**
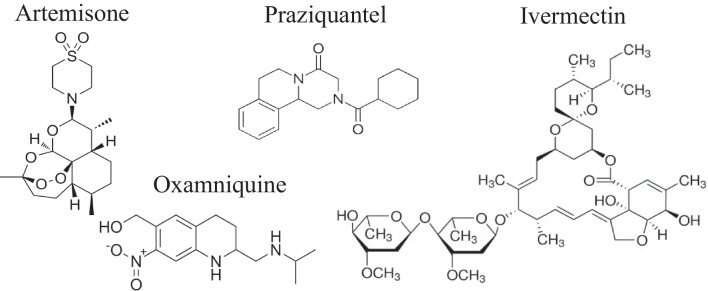
Chemical structures

The current chemotherapy and prevention of schistosomiasis rely on one drug, praziquantel. An alternative oxamniquine was previously used only against *S. mansoni* in cases of failure of the therapy with praziquantel. Oxamniquine (Scheme 1) is active, owing to DNA binding, but it affects only mature parasites and it occasionally provokes serious side effects. However, resistance of *S. mansoni* to oxamniquine has been demonstrated both in the laboratory and in the field (Da Silva et al. 2017) and consequently, new oxamniquine derivatives have been proposed as a future alternative (Alwan et al. 2023). Praziquantel (PZQ, Scheme 1) is recommended as the drug of choice for the treatment of schistosomiasis due to its safety and effectiveness against all major forms of schistosomiasis. It was suggested that PZQ interacts with calcium regulation in the parasite membrane, causing irreversible damage to adult worms. Mammals are not affected due to differences in the relevant receptors (Cunha and Noël, 1997; Chulkov et al. 2023; Park et al. 2021). Due to the massive use of PZQ, there is a constant selective pressure towards establishment of resistance (Le Clec’h et al. 2021). Indeed, low cure rate, reduced susceptibility of *S. mansoni* to PZQ, and treatment failures have been reported, raising concerns about PZQ resistance and drug efficacy (Vale et al. 2017; Caldwell et al. 2023). Moreover, “The pipeline is nearly empty and no other therapeutic alternative has reached the market” (Spanbenberg 2021); there are no approved alternatives to PZQ.

Drugs that are already approved for human use have obvious advantages in searching for new therapeutic indications, including reduced costs and timelines because some routine steps of drug development and regulations are not required. Some drugs were suggested for treatment of schistosomiasis based on repurposing ideas (Spanbenberg 2021; Roquini et al. 2023), mostly artemisinins, their derivatives, and formulations (Keiser and Utzinger 2012; Zech et al. 2020). A search of compounds repurposed for treatment of schistosomiasis (“Repurposing drug schistosomiasis/bilharzia”, PubMed January 23, 2024) shows 64 publications. The majority of these drugs/formulations are not (yet?) approved for human use and have not been examined in vivo; the conclusions concerning their possible use are mostly based on in vitro effects in worm membrane and tegumental integrity (e.g., Beutler et al. 2023). Of these publications, a few deal with the potential use of ivermectin for treating schistosomiasis (e.g., Keiser and Utzinger 2012; Moreira et al. 2022; Siddiqui et al. 2021). The suggestions concerning ivermectin for treatment are also mainly based on in vitro studies. The relatively high concentration of ivermectin used in these studies makes it impossible to pharmacologically achieve the dose required for significant activity, since the dose is probably higher than the maximum plasma concentration of ivermectin achieved in vivo (Ryan et al. 2023).

The avermectins are a series of drugs and pesticides used to treat parasitic worms and insect pests of which ivermectin is the most effective. Some publications consider ivermectin for treatment of schistosomiasis (Taman et al. 2014; Siddiqui et al. 2021; Ryan et al. 2023). Ivermectin (Scheme 1) is a macrocyclic lactone that has been approved as an anti-helminthic drug especially for treatment of filarial infections. It is a selective positive allosteric modulator at the glutamate-gated chloride channels that are found in nematodes, insects, and other invertebrates and acts by binding to these channels leading to chloride ion influx causing hyperpolarization of the cell and hence dysfunction (Martin et al. 2021). Ivermectin is essential in the success of the control of *Onchocerca volvulus*, the causative agent of river blindness, and is also applied against other parasitic nematodes and various ectoparasites (Brattig et al. 2021; Kositz et al. 2022). Ivermectin also potentiates other chloride channels, including the ones gated by GABA. Mammals ordinarily are not affected because they lack glutamate-gated chloride channels, and there is a lower drug affinity for other mammalian chloride channels. It is valid against nematodes and some invertebrates but it does not influence cestodes and trematodes—ivermectin acts as the GABA receptor agonist, and cestodes and trematodes lack a GABA system (Feng et al. 2002).

Nevertheless, ivermectin was suggested for treatment of schistosomiasis because of the following reasons: (a) ivermectin has a general immunosuppressive activity (Blakley and Rousseux 1991; Piras et al. 2022) and a more specific activity against schistosomes that is related to inflammation: ATP-gated P2 receptors are associated with a signaling pathway linked to the liver and mesenteric exacerbations of schistosome-related inflammation. Therefore, it has been proposed that members of this purinergic signaling could be putative pharmacological targets that may reduce schistosome morbidity which is associated with inflammation (Oliveira 2021) or cancer (Siddiqui et al. 2021). (b) Interference with VEGF angiogenic cascade that is essential for schistosome survival (Shariati et al. 2011; Siddiqui et al. 2021). (c) A potential fringe benefit of ivermectin that is massively used for treatment of filarial infections in areas that are endemic for both filariasis and schistosomiasis. Apparently, ivermectin may also serve as an anti-molluscan agent, affecting the intermediate hosts of schistosomes (Katz et al. 2017).

A few clinical trials were performed to evaluate the efficacy of ivermectin in human patients infected with schistosomes, mostly using drug combinations. A single dose given to patients with different concomitant infections yielded a mild effect on *S. haematobium* infection (Makunde et al. 2000 (150 mg); Agere et al. 2014 (40 mg) and no effect on *S. mansoni* infection (Whitworth et al. 1991 and Njoo et al. 1993 (150 mg)). Few experiments were conducted in animal models either with no- or partial success (detailed in the discussions).

Overall, the current literature is not clear enough to confirm or deny a positive role of ivermectin in *Schistosoma* treatment. The success of in vitro experiments does not necessarily predict in vivo significance. Consequently, we examined the efficiency of ivermectin in a demanding manner: high drug concentrations (still tolerable for short periods, throughout the experiments) in infected mice by delivery in different days (up to four treatments following infection).

## Materials and methods

### Ivermectin

Ivermectin was purchased from Sigma (Israel) and was used after dilution in DMSO. In some experiments, we used Ivomec (Merial) where ivermectin is diluted in glycerol and propylene glycol.

We used ivermectin in 125 µl DMSO/treatment and acute toxicity was not observed throughout the experiments. The gavage of ivermectin in 100 µl of Ivomec (that does not contain DMSO) yielded similar results.

### Artemisone

Artemisone was donated by CIPLA, India. It was dissolved in DMSO and used as a positive control based on its significant effect in mice infected with *S. mansoni* (Zech et al. 2020).

### Schistosoma mansoni

Experiments were performed using the Puerto Rican isolate obtained from NIH. The life cycle of *S. mansoni* was maintained in ICR mice and *Biomphalaria glabrata* snails. The snails were raised and kept at 26 °C in aerated aquaria. Mice were routinely infected by subcutaneous injection of about 150 cercariae each. Seven to 8 weeks postinfection, schistosome eggs were extracted from the granuloma-containing livers and hatched by exposure to light. *Biomphalaria glabrata* snails were infected individually by exposure to 7–8 miracidia each. Cercariae were obtained from infected snails by exposure to light for 1–2 h, starting 4 weeks after snail infection.

### Mice

Male ICR mice were purchased from Harlan Laboratories (Rehovot, Israel). These mice were used for the schistosome infections and treatment at Tel-Aviv University (Tel Aviv University ethical committee number 01–13-076). The mice were infected by subcutaneous injection with 150 cercariae.

### Treatment of mice infected by* Schistosoma mansoni*

ICR male mice, *n* = at least 5/group, 7–8 weeks old, 32–35 g at infection, were treated postinfection (PI) by oral gavage of ivermectin 30.1 mg/kg in 125 µl of DMSO on various days postinfection. In some experiments, we treated mice by oral gavage of identical ivermectin concentrations in 100 µl Ivomec. Artemisone 40 mg/kg was used as a positive control (Zech et al. 2020). 125 µl DMSO was used as a control in gavage.

### Assessments of treatment success

Schistosome numbers were determined by counting worms from dissected and squashed livers and mesenteric veins of dissected intestines of the mice, 49–51 days PI by using a binocular stereomicroscope. In parallel, liver samples of mice were fixed in 4% formalin (v/v) and paraffin-embedded; 4-µm sections were cut and stained with H&E for estimating granuloma density and area. Granuloma number was calculated/area (estimated in large scale images).

Each type of experiment was repeated at least once. The significance of results was estimated by *T* test of Prism.

### Experimental design

The experiments were conducted in three stages:

- a. Infection of mice with *S. mansoni* cercariae.
- b. Gavage with ivermectin or artemisone at various intervals following infection.
- c. Assessment of the results of the treatment (49–51 days PI) by determining worm burden and in parallel examining liver pathology.

## Results and discussion

Ivermectin or artemisone was delivered to infected mice by gavage at different periods postinfection (PI). Table 1 summarizes the results.

**Table 1:** Summary of drug effects on schistosome development

Compound	Day of treatment	Control worm number	Worm number post treatment	Significance
Ivermectin	21	22.8 ± 3.1	22.0 ± 4.6	NS
	21, 22	45.6 ± 6.7	43.5 ± 9.5	NS
	21, 28	12.5 ± 3.3	18.3 ± 3.0	NS
	21, 42	22.8 ± 3.1	33.1 ± 4.2	NS
	*21, 22, 41, 42	20.1 ± 3.3	32.2 ± 3.8	NS
	41, 42	45.6 ± 6.7	36 ± 7.1	NS
Artemisone	21, 28	19.8 ± 2.9	3.5 ± 1.4	S
	41, 42	19.8 ± 2.9	1.8 ± 0.2	S

*NS* not significant, *S* significant *p* < 0.05

*An experiment applying gavage of Ivomec (see “Materials and methods”) in the same ivermectin concentration revealed similar results: —the ivermectin had no effect

**Fig. 1:**
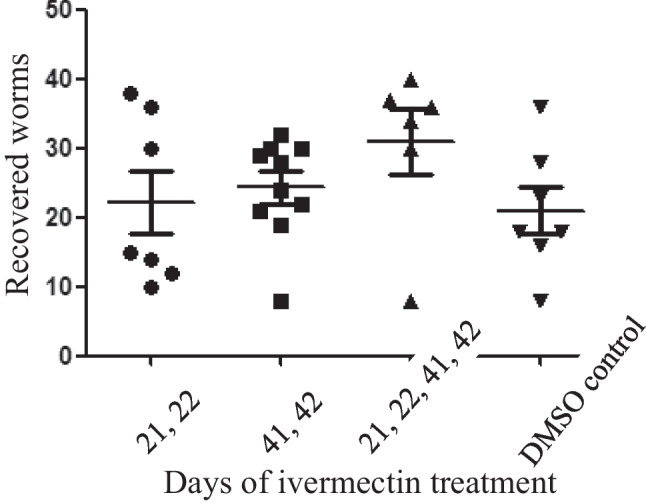
Effects of ivermectin delivered by gavage on different days postinfection

All infected mice were examined (number of adult worms and histology) on days 49–51 postinfection (PI). Ivermectin treatment (30.1 mg/kg) by gavage at different intervals PI did not affect neither the number of adult worms nor the female/male ratio (about 1:1) and the size and the number of liver granulomas. The timing of treatment, early or late in the course of infection, did not affect the results. In contrast, the positive control of artemisone (40 mg/kg) was effective as expected. Artemisone significantly reduced the number of adult worms and the effect was slightly more pronounced in mice treated at a later stage of the infection. A representative experiment is presented (Fig. 1), indicating the lack of ivermectin effect on worm burden.

The infected mice were examined (number of adult worms and histology) on days 49–51 postinfection. Each point represents a single mouse. Shown are the numbers of adult worms (males and females) and the standard errors.

**Fig. 2:**
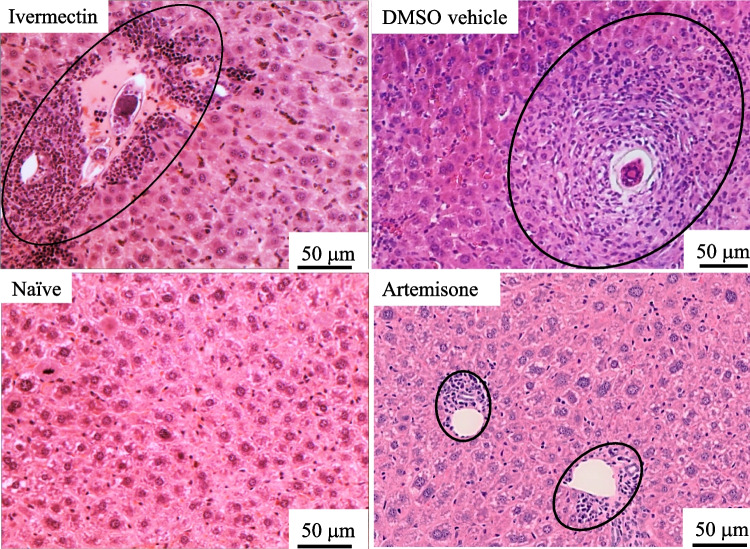
Liver histology of schistosome-infected mice treated with ivermectin or artemisone on days 41 and 42 postinfection

**Fig. 3:**
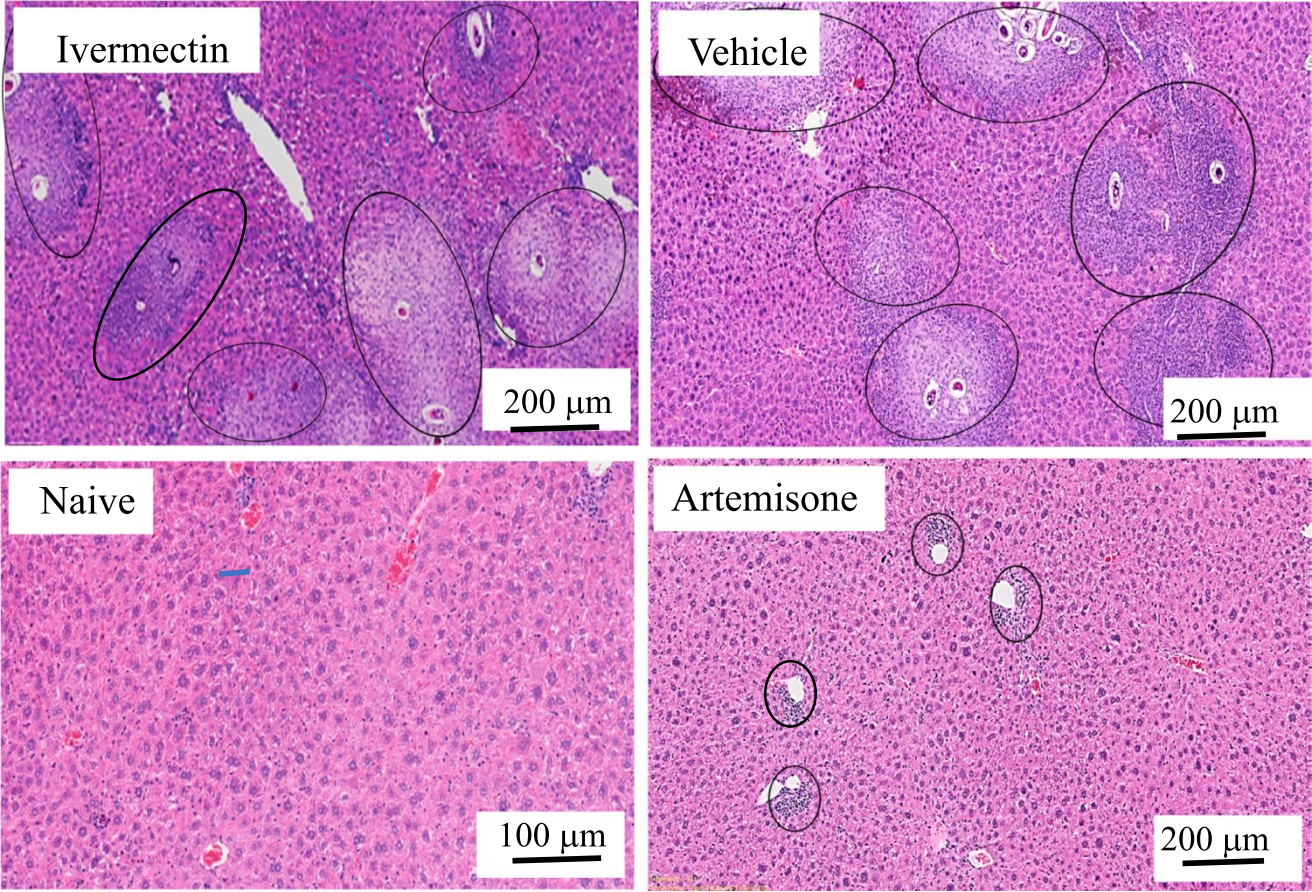
Liver histology of schistosome-infected mice treated with ivermectin or artemisone on days 21, 22, 41, and 42 postinfection

**Fig. 4:**
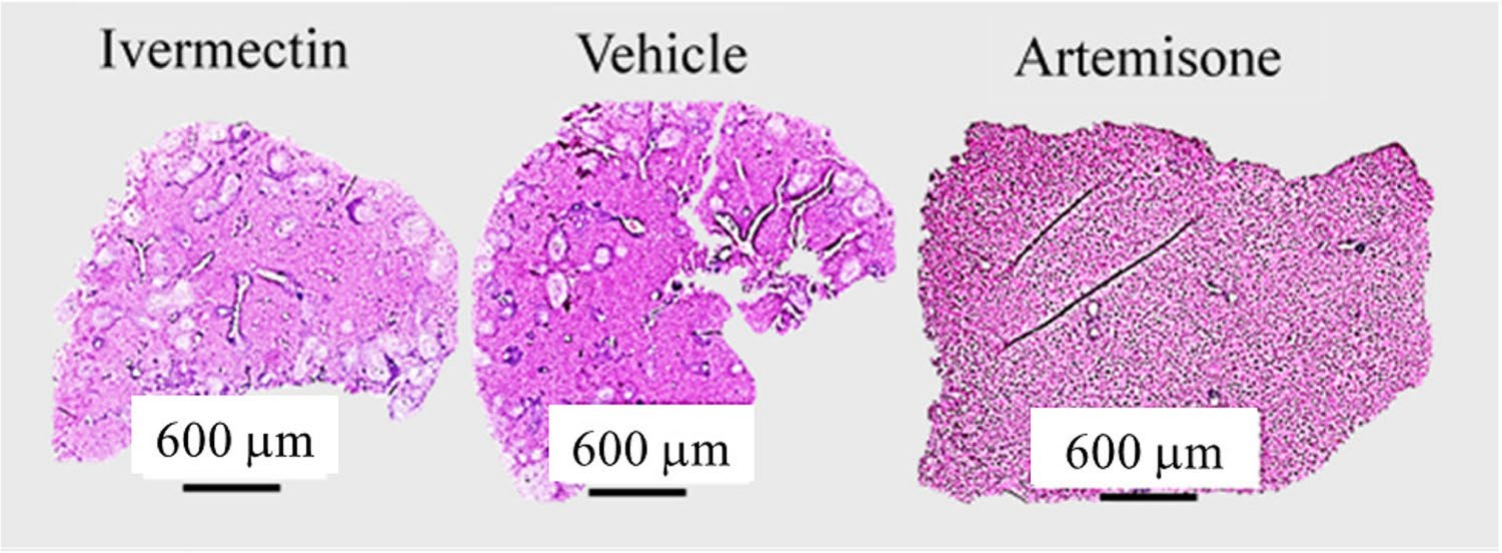
Liver transverse sections of schistosome-infected mice treated with ivermectin, DMSO vehicle, or artemisone on days 21, 22, 41, and 42 postinfection

Drugs (30.1 mg/kg) were delivered by gavage on different days. Ivermectin did not reduce the infection severity, neither the worm number (*p* > 0.05) nor the estimated number and shape of granulomas regardless of the days of treatment. Representative liver sections demonstrate lack of effect of ivermectin in contrast to artemisone (Figs. 2, 3, and 4).

The photographs shown in Fig. 2 are liver histology sections of representative naïve and schistosome-infected mice treated by vehicle, ivermectin, or artemisone. The mice were treated by gavage with drugs (30.1 mg/kg) on days 41 and 42 PI.

The photographs shown in Fig. 3 are liver histology sections of representative naïve and schistosome-infected mice treated by DMSO vehicle, ivermectin, and artemisone. The mice were treated by gavage with the drug (30.1 mg/kg) or vehicle on days 21, 22, 41, and 42 PI.

There was no difference between the effects of the drug and the vehicle: the mean size ± se for ivermectin treatment is 17.0 ± 2.2 vs. 21.2 ± 2.9 area units for DMSO control (*p* = 0.3).

The photographs shown in Fig. 4 were processed by amplifying the contrast in order to enable a more accurate estimation of the number and size of granulomas. There was no difference in granuloma nuber and size between the ivermectin and DMSO treatment. The artemisone prevented granuloma formation.

In summary, we used ivermectin by oral gavage in schistosome-infected mice, at different doses, up to a bordering toxicity (30.1 mg/kg); however, there was no immediate toxicity throughout the experiments. Ivermectin was delivered at various intervals PI to check on possible effect at different stages of parasite development but none of the treatment regimens resulted in cure rates or signs of lessened pathology in the mice. Both, ivermectin and vehicle treatments did not influence worm number, granuloma size, and formation around schistosome eggs (central in granulomas).

We compared the effect of ivermectin to that of artemisone, an artemisinin derivative which successfully had served us in the past (Zech et al. 2020) as an effective anti-schistosome agent; there was a stark difference in artemisone’s efficacy compared to that of ivermectin: while the ivermectin was not effective, the artemisone eliminated the worms, prevented egg production and granulomatous inflammatory response (Table 1, Figs. 2, 3, and 4). In a minority of artemisone-treated mice (about 25% of artemisone-treated animals), there was a background inflammatory response near sinusoids. This may occur because of the abundance of schistosome antigens following the treatment (Reimers et al. 2015; Zech et al. 2020). Likewise, treatment of worms with an anti-helminthic drug may often cause a massive release of antigens that induces a serious allergic response (Satti et al. 2004). Anyhow, we did not observe an immediate abnormality in the artemisone-treated mice.

The only drug of choice against schistosome infections is PZQ. It is a successful drug because of its selective activity based on activation of a TRPM_PZQ_ permeable Ca^2^ ion channel that induces paralysis and consequently increases sensitivity of the parasite to host immune responses (Brindley and Sher 1990; Chulcov et al. 2023; Waechtler et al. 2023). Emerging resistance (Aruleba et al. 2019) and occasional serious allergies (Satti et al. 2004) following PZQ therapy increased the search for alternative drugs. Various approved compounds were examined by using several methods in an attempt to increase the repertoire of anti-schistosome drugs and exploit the advantages of drug repurposing, hoping to find a differential effect on schistosomes and their animal hosts. Docking to target molecules, in vitro and in silico screening and examinations in animal models were also used to examine ivermectin. Despite the differences in the mode of action of PZQ and ivermectin, both have allegedly a wide scope of activities including effects on various receptors that are vital to transport and anti-inflammatory activity (Nogueira et al. 2022; Johnson-Arbor 2022; Chen and Kubo 2018). The expected obvious benefits of repurposing and these shared similarities initiated the idea of examining ivermectin as an anti-schistosome drug. In addition, it was speculated that due to its wider range of ligand-gated channels found in invertebrates, ivermectin will be useful in prevention and treatment of schistosomiasis (Laing et al. 2017).

A literature screening reveals only few investigations that examine in vivo effects of ivermectin on schistosomes. The conclusions are mostly analogous to those relating to the applicability of ivermectin for COVID-19 therapy (Shafiee et al. 2023): in both cases, there were in vitro significant results versus in vivo lack of success. Likewise, despite the pronounced effect on worm motility, there was no reduction of worm burden in infected mice following gavage treatment of 25 mg/kg ivermectin in 2 days, starting 6 weeks postinfection (Ryan et al. 2023). A failure of prophylaxis attempts (1 mg/kg delivered by gavage in three successive days before infection) was observed in infected mice (Vicente et al. (2021). In contrast, there was a slight (but statistically significant) reduction in worm burden, destructive changes (mainly in female worms), decreased liver inflammation, and an increase in the quantity of dead ova in mice that were treated with 25 mg ivermectin/kg by gavage, in two consecutive days starting 6 weeks PI (Taman et al. 2014).

Some publications hint to an absence of effect of ivermectin treatment on schistosome infections in people living in the tropics: ivermectin massive treatment of onchocerciasis did not induce significant short-term changes in *S. mansoni* egg count in stool specimen of inhabitants living in an identical endemic area (Whitworth et al. 1991; Njoo et al. 1993). Njoo et al. examined patients treated once with ivermectin just 3 days posttreatment, and Whitworth et al. examined ivermectin effect 45–105 days posttreatment. Unfortunately, these studies were not designed for proper estimation of ivermectin effect on schistosome; despite the feasible benefit of examining drugs in human patients, the studies ignore the possible effect of various concomitant infections on each other.

DMSO (Huang et al. 2020) and ivermectin (Sajid et al. 2007) have a known immunomodulatory effect. Theoretically, immunomodulation could be an efficient measure against the immunopathology that is demonstrated in schistosomiasis. However, in our experiments, ivermectin (that was dissolved in DMSO), DMSO, and ivermectin in Ivomec (that does not contain DMSO) had similar effects. Anyhow, schistosomes themselves have a variety of immunomodulators that hypothetically enable their in vivo existence during many years (Acharya et al. 2021). Interestingly, there are reports relating to the use of schistosome-derived products as modulators, for the prevention and alleviation of immunological disorders such as auto-immune diseases (Mu et al. 2021).

Probably, the lack of activity of ivermectin especially in comparison with PZQ and artemisinins originates from the reported difference in their mode of action: ivermectin acts as the GABA receptor agonist; however, cestodes and trematodes lack a GABA system (Feng et al. 2002) and PZQ targets specifically the schistosome TRPM_PZQ_. The detrimental effect of artemisone on malaria and schistosomiasis follows oxidative stress, a consequence of reaction to blood-dwelling parasites (Zech et al. 2020). Iron-dependent and -independent reaction pathways of artemisinins are related to perturbation of redox homeostasis that ultimately leads to generation of deleterious reactive oxygen species (Meshnick 1994; Quadros et al. 2022).

We cannot explain the differences between the total lack of ivermectin effect in our hands vs. that of Taman et al. (2014) despite the fact that we used ivermectin at a concentration even exceeding their treatment. There are of course some differences in parasite strain (local Egyptian vs. ours– Puerto Rican) and strains of mice, as also the number of cercariae used for infection. However, it is hard to reconcile the differences in the results between our group and theirs. Interestingly, Barda et al. (2016) demonstrated the success of other macrocyclic lactones in reducing schistosome number in rodents. However, despite the positive results, drug derivatives must be approved for use by applying the usual complicated and long procedure towards human use.

## Conclusions

Because of some instances of PZQ resistance in schistosome infections and similarities in the scope of activities of PZQ and ivermectin, there have been attempts to employ ivermectin as a possible alternative. In wake of our experiments with ivermectin and *S. mansoni*-infected mice and despite the results of Taman et al., we conclude that ivermectin as such is not an alternative to PZQ, in contrast to artemisinins that might replace or add (Dong et al. 2014) to PZQ due to their efficient mechanism of action.

## Data Availability

Data is available upon request.
